# Optical Methods to Study Protein-DNA Interactions *in Vitro* and in Living Cells at the Single-Molecule Level

**DOI:** 10.3390/ijms14023961

**Published:** 2013-02-18

**Authors:** Carina Monico, Marco Capitanio, Gionata Belcastro, Francesco Vanzi, Francesco S. Pavone

**Affiliations:** 1 European Laboratory for Non-linear Spectroscopy, Via Nello Carrara 1, 50019 Sesto Fiorentino (FI), Italy; E-Mails: capitan@lens.unifi.it (M.C.); belcastro@lens.unifi.it (G.B.); fvanzi@lens.unifi.it (F.V.); pavone@lens.unifi.it (F.S.P.); 2 Department of Physics and Astronomy, University of Florence, Via Sansone 1, 50019 Sesto Fiorentino (FI), Italy; 3 Department of Evolutionary Biology “Leo Pardi”, University of Florence, Via Romana 17, 50125 Florence, Italy; 4 National Institute of Optics–National Research Council, Largo Fermi 6, 50125 Florence, Italy; 5 International Center of Computational Neurophotonics, Via Nello Carrara 1, 50019 Sesto Fiorentino (FI), Italy

**Keywords:** single-molecule techniques, protein-DNA interactions, facilitated diffusion, tethered particle motion, optical tweezers, magnetic tweezers, DNA curtains, single-molecule imaging, combining single-molecule fluorescence and optical trapping, *in vivo* transcription factors dynamics

## Abstract

The maintenance of intact genetic information, as well as the deployment of transcription for specific sets of genes, critically rely on a family of proteins interacting with DNA and recognizing specific sequences or features. The mechanisms by which these proteins search for target DNA are the subject of intense investigations employing a variety of methods in biology. A large interest in these processes stems from the faster-than-diffusion association rates, explained in current models by a combination of 3D and 1D diffusion. Here, we present a review of the single-molecule approaches at the forefront of the study of protein-DNA interaction dynamics and target search *in vitro* and *in vivo*. Flow stretch, optical and magnetic manipulation, single fluorophore detection and localization as well as combinations of different methods are described and the results obtained with these techniques are discussed in the framework of the current facilitated diffusion model.

## 1. Introduction

At the most elemental level, all DNA biological functions are carried out by individual proteins that must interact with DNA (usually with specific sequences) to trigger molecular processes indispensable to the cell. Some examples include DNA replication, gene expression and its regulation, DNA repair, genome rearrangement by DNA recombination and transposition, as well as DNA restriction and modification by endonucleases and methyltransferases, respectively. A myriad of reasons make the study of protein-DNA interactions underlying these processes very captivating from both the biological and biophysical points of view. First, DNA is confined in a cellular compartment (in the nucleus in eukaryotic cells or nucleoid region in eubacteria), and the accessibility of DNA sequences to proteins is further restricted by the supercoiled structure of native DNA in eubacteria or by nucleosomes in eukaryotic chromatin. Moreover, to find specific binding target sequences and perform their activities, proteins must deal with the crowded environment of the cell and with the presence of roadblocks along the DNA chain (such as higher-order chromatin structures, nucleosomes, or other DNA-binding proteins). In fact, the mechanism by which proteins are able to find relatively small cognate sequences (typically 15–20 bp for repressors [1] and 4–6 bp for restriction enzymes [2]) amongst the millions of base pairs of non-specific chromosomal DNA has been puzzling researchers for decades [3]. Further, the complexity of the target search process is enhanced by the fact that many DNA-binding proteins (for example transcription factors) are typically present at levels of only a few copies per cell [4,5]. The fundamental regulatory role of many of these proteins, on the other hand, requires them to be able to locate their cognate sequences, activate or repress genes depending on the cell needs, and adapt to environmental changes on reasonable time scales [6–8].

Experimental attempts to elucidate protein-DNA interactions started a few decades ago. Historically, one of the most studied transcription factors is the *E. coli* lactose repressor protein (LacI [9]). In 1970, Riggs and coworkers measured the association rate of LacI to its operator site as *k_on_* = 7 × 10^9^ M^−1^ s^−1^ [10]. This surprisingly high association rate evoked particular interest. First because it was about three orders of magnitude higher than other known biological binding rates, such as protein-protein interactions with typical association rates in the order of 10^5^–10^6^ M^−1^ s^−1^ [11]. Second, it was almost 100 times faster than the maximal rate of association estimated for pure 3D thermal diffusion and random collision for a protein in the cytoplasm (~10^8^ M^−1^ s^−1^ [12–14]). Although low ionic strength buffer could have contributed to an enhancement of the measured association rate [15], those findings triggered several kinetic and theoretical studies that investigated this seemingly faster-than-3D diffusion binding of proteins to DNA. As a result of these studies, the facilitated diffusion model [3,16–21] is currently the most accepted theory of DNA target searching, and it has been recently demonstrated *in vivo* for LacI [22]. In this model, a protein diffuses in 3D until a random collision with a DNA segment leads to a non-specific interaction. The protein then switches to 1D diffusion along the DNA, until it either encounters a specific target or simply dissociates from DNA due to its finite dissociation constant [6,14,23,24]. In this model the faster-than-3D diffusion binding is thus explained by a reduction in the dimensionality of the search process, which, for some interval of time (during the sliding), is reduced from 3D to 1D. The exploration of non-specific DNA by sliding therefore plays a very important role in accelerating cognate site search. The relative times spent in 3D and 1D diffusion determine the impact on the effective association rate, and it has been found that the optimal rate is obtained for short sliding distances (~50 bp [15]).

**Figure 1:**
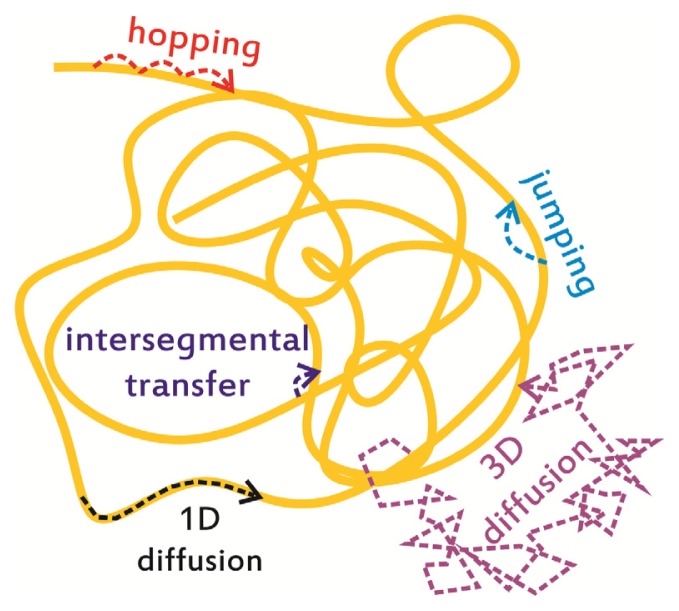
Several mechanisms are involved in target search by site-specific DNA-binding proteins: 3D diffusion in the cell, 1D sliding along DNA, hopping and jumping, as well as intersegmental transfer.

In a general description of all the potential processes occurring during facilitated diffusion (Figure 1), the terms “hopping” and “jumping” are often used. In these cases, after the 1D search phase, the protein momentarily loses contact with the DNA before reassociating with a new site in the vicinity of the previous, thus maintaining a close proximity to the explored positions. The distinction between “hops” and “jumps” is still somewhat arbitrary, although the term hopping usually describes short (<10 bp) successive steps and jumping corresponds to larger steps (>100 bp) along the DNA molecule [8,15], as depicted in Figure 1. A further mechanism is named “intersegmental transfer”, which is only relevant for DNA-binding proteins with more than one binding site (such as LacI [25] and some restriction enzymes [26]). During intersegmental transfer, the protein moves between two sites via an intermediate looped state in which the protein is interacting with both sites simultaneously.

The complex interplay of all these processes determines the rate at which a protein can scan through the excess non-specific DNA and find the needle in the haystack, *i.e.*, its target. Some of these processes, for example intersegmental transfer, require specific structural features such as the ability of binding simultaneously to multiple sites, but all of the most basic properties of the search process can be described in terms of association and dissociation rate constants (as distinguished between those relative to specific and non-specific DNA) and diffusion constants (both 3D and 1D). Once a protein undergoing free 3D diffusion, characterized by its *D*_3D_ diffusion constant, collides with DNA and interacts with it (occurring statistically most often with non-specific DNA), it will undergo two competing processes: sliding along the DNA, characterized by *D*_1D_ diffusion constant, or dissociation, characterized by an off rate. The equilibrium between these two competing processes determines the average distance scanned by the protein along the DNA before dissociating and resuming free 3D diffusion. The measurement of the fundamental kinetic properties of the protein is the basis for understanding its target search mechanism. It can be easily understood that some of these parameters crucially depend on the DNA sequence, which determines the interaction energy landscape the protein explores in its interactions and during sliding along the DNA, and on DNA conformation and occupancy by other proteins. Thus, a complete picture describing these processes in the cell requires measurements in which each of these variables is independently controllable.

The enhanced efficiency of target location by this ensemble of mechanisms becomes more intuitive if one considers for example the bacterial native structure of DNA. Considering the DNA persistence length (~150 bp), any DNA molecule in the cell behaves as a random coil and native DNA it is typically supercoiled or compacted into chromatin, which can facilitate the juxtaposition of distal sites along the DNA chain [27].

As mentioned above, studies of these processes including theoretical modeling of the underlying mechanisms started as early as the 1970s and were developed mostly through conventional bulk biochemical measurements. In the last decade, the biophysical characterization of protein-DNA interactions and target search mechanisms has gained further momentum with the use of single-molecule methods. Among several advantages with respect to traditional bulk experiments, in which the behavior of individual proteins is obscured by ensemble averaging, single-molecule techniques permit probing of the dynamics of single biomolecules in real time [28–30]. Recently, single-molecule optical methods have witnessed striking progress in reaching high sensitivity and resolution, demonstrating their value as an effective tool to study biology. For example, single-molecule manipulation techniques such as optical tweezers make it possible to probe DNA conformations and mechanical properties by precisely controlling the end-to-end distance of DNA molecules. Furthermore, the application and measurement of mechanical loads that affect energy landscapes of protein-DNA interactions have become common practice. Additionally, single-molecule imaging techniques allow detection of single fluorescently-labeled biomolecules and their localization with a precision of few nanometers [31]. Hence, it is now possible to reconstruct a protein trajectory by determining its position with high precision in time and space.

This review describes the main contributions of single-molecule optical techniques to our current understanding of protein-DNA interactions, including a discussion of the state-of-the-art methodologies developed for both *in vitro* and *in vivo* experiments.

## 2. *In Vitro* Monitoring of Facilitated Diffusion and Protein-DNA Interactions at the Single-Molecule Level

### 2.1. Tethered Particle Motion

The tethered particle motion (TPM) assay, introduced by Jeff Gelles and coworkers at the beginning of the 90s [32,33], is well-suited to probe the dynamics of DNA binding proteins which cause bending, shortening or looping of DNA strands. These DNA manipulation properties are shared by regulatory proteins of DNA metabolism and restriction enzymes, among others [34]. The study of DNA looping kinetics is thus of great interest and it has been extensively characterized by TPM assays as exemplified below.

**Figure 2:**
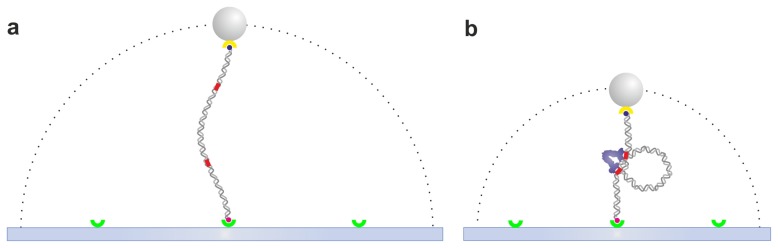
The tethered particle motion assay. (**a**) A single DNA molecule is tethered between the slide surface and a polystyrene bead or gold nanoparticle through specific binding, such as streptavidin-biotin and digoxygenin/antidigoxygenin. The dotted line represents the allowed range of diffusion of the microsphere, which depends on the DNA contour length. (**b**) Lactose repressor protein (LacI)-DNA interaction. LacI (blue) binds simultaneously to the two operator sequences (red) and induces a loop, thus shortening the DNA tether. Drawings are not to scale. Throughout the paper all figures are drawn on a scale chosen to emphasize the details of the biological molecules and relevant protein-DNA interactions. On this scale, the microspheres (typically in the 0.2–2.0 μm range) would be about 40 times larger than drawn.

The TPM assay consists of tethering a microsphere to the microscope coverslip through a single DNA molecule as depicted in Figure 2. The diffusion of the bead by Brownian motion is constrained to a hemispherical region by the DNA molecule. By tracking the range of allowed mobility of the bead with Differential Interference Contrast (DIC) microscopy [32,35], Total Internal Reflection Fluorescence (TIRF) microscopy [36], darkfield microscopy [37] or simply by transmission (bright field) microscopy [38,39], one can detect changes in DNA length induced by looping in real-time. The simplicity and adaptability of TPM to different biological systems makes this technique broadly applicable. A simple commercial optical microscope is sufficient to get access to single-molecule recordings of protein-DNA interaction using the TPM assay. More sophisticated but still easily accessible techniques, such as DIC, TIRF or darkfield microscopy, allow the use of smaller probes. TPM has been used to monitor the processivity of single RNA polymerases transcribing DNA [32,33], LacI-mediated DNA loop formation and breakdown [38–41], as well as to determine the effect of DNA length [42,43], sequence [44], or forces [45,46] on LacI-mediated looping. Other examples include the study of IS911 DNA transposition [47], DNA-looping dynamics by lambda repressor [48,49], restriction enzymes with two binding sites, such as NarI and NaeI [39] and FokI [50], the bending pathway of promoter sequences by TATA-box binding protein [51], as well as lambda [52] and Cre-mediated [53] site-specific recombination pathways.

The application of flow to the DNA-bead complex creates a different geometry, resulting in an improved spatial/temporal resolution due to the suppression of the bead Brownian motion [54]. Moreover, to increase the throughput of TPM experiments, Salomé’s group recently developed a biochip to monitor hundreds of protein-DNA complexes in parallel [55].

Technically, TPM is a very simple method to implement, requiring only a microscope equipped with a camera for video recording. The data analysis methods are also simple and can be based on either centroid tracking [38] or frame averaging and measurement of the averaged bead position distribution [40]. Perhaps a more delicate aspect of this method regards temporal resolution [56] and data analysis: since all analysis approaches require time averaging, proper methods need to be adopted to obtain reliable kinetic parameters from TPM measurements [57–59]. Another notable issue involves the steric effects of the tethered microsphere, which have been investigated both theoretically [60] and experimentally [61].

Compared to the methods illustrated below, TPM has the limitation of being applicable to proteins that cause detectable length changes in the DNA molecule (typically looping, but also DNA compaction or bending). In the case of sliding, the protein must be immobilized to the surface in order for its mechanical activity to be detected through bead mobility, but surface immobilization is often detrimental to the enzyme activity. In this regard, other more complex techniques illustrated below offer a better option. Another disadvantage of TPM is the low time resolution, which is limited by the time required by the probe to explore the hemispherical region allowed by the DNA tether [59]. A very detailed description of the methods used for TMP experiments can be found in all articles cited above, particularly in [33] and [38].

TPM experimental design allows a straightforward combination with optical and magnetic tweezers. The latter techniques enable the application of controlled forces or torques on the biological molecules under study.

### 2.2. Optical Tweezers

**Figure 3:**
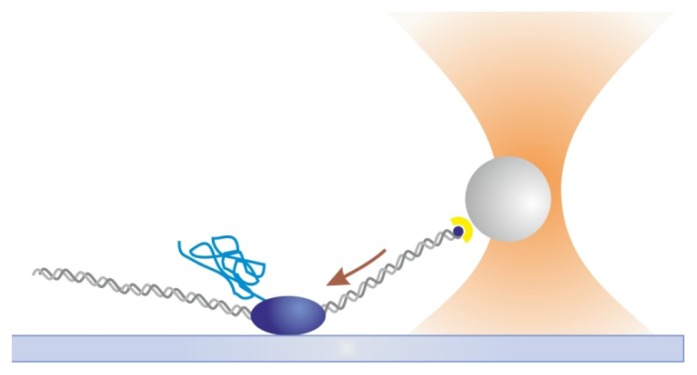
Single optical tweezers assay. A single RNAp is attached to the slide surface and the 3′ end of the DNA template is attached to an optically trapped bead. As the DNA is transcribed, the RNAp pulls the bead in the direction shown by the arrow. Drawing not to scale.

Optical tweezers (OT) [62–64] are formed by tightly focusing a laser beam to a diffraction-limited spot with a high numerical aperture lens, such as a microscope objective. They act as true “tweezers” because dielectric particles such as polystyrene or silica beads, to which DNA or the protein of interest is attached, can be stably trapped and displaced within the sample chamber. In OT-based assays, pN-range forces can be measured, applied and actively clamped, using for example PID algorithms [65], to study molecular interactions under load. Several experimental configurations can be used in the OT assay, as described in this manuscript. The implementation of OT to *E. coli* RNA polymerase (RNAp), allowed Wang and co-workers to measure transcription velocity as a function of force at the single-molecule level [66] (Figure 3). Loads of up to 25 pN could be applied without disrupting transcription. This load value is defined as the stalling force of RNAp, *i.e*., the maximum force that can be produced by RNAp, reflecting a tight binding of the polymerase during the elongation cycle of transcription. Later on, Steven Block and colleagues worked on a different configuration to pull on the nascent RNA molecule to probe the effects of folded RNA hairpins on RNAp processivity, transcription elongation rates and pausing [67]. The single OT assay was also used to probe the helical movement of RNAp corresponding to the groove of DNA while searching for promoter sequences [68].

Wang and colleagues developed an experimental assay to study several protein-DNA interactions [69] based on the mechanical unzipping of single DNA molecules [70,71]. Briefly, one strand of the DNA is anchored to the microscope coverslip and the other strand is attached to a microsphere held in the OT. The DNA is unzipped as the microscope coverslip moves horizontally away from the optical trap. The analysis of the unzipping forces and DNA tether length reveals information about the location of bound proteins and equilibrium association constants [69]. This unzipping force analysis was implemented to study restriction enzymes [72], DNA-repair proteins [73], high-resolution mapping of interactions between nucleosomal DNA and core histones [74], and the unwinding of dsDNA by T7 helicases [75,76]. The single OT assay was used to measure the motion and sequence-dependent pausing of lambda exonuclease (an ATP-independent processive enzyme that degrades dsDNA to create single stranded overhangs used in recombination by the bacteriophage lambda) [77], the unwinding and backward motion of RecBCD (a bacterial DNA helicase and nuclease responsible for resection of double stranded DNA) [78], and the binding strength between DNA and histones in a nucleosome array [79], among others.

Building an OT setup requires expertise in optics and specific knowledge of single-molecule techniques. OTs are usually implemented by inserting an infrared laser beam in the optical path of a modified commercial optical microscope. The collimated beam is focused by a high numerical aperture objective to create the optical trap and then collected by the microscope condenser to be projected on a quadrant detector photodiode for position detection, although many variations on the detection scheme can be found. Inserting piezo, galvo or motorized mirrors, acousto or electro-optic deflectors or spatial light modulators can provide fine control over trap displacements or multiple tweezers through time-sharing [80,81]. A long list of prescriptions and precautions must be taken into account to minimize mechanical noise [82,83] and to guarantee accurate calibration and measurements [84,85]. OT setups, although widely used, therefore remain specialized tools. Several reviews summarize the technical aspects of building such setups [86,87].

Overall, optical tweezers enable the application of controlled loads to the DNA tether or to the protein interacting with DNA and therefore the investigation of force-dependent kinetic rates. Moreover, special technological advances permit the development of high spatial resolution OTs [88], capable of resolving the single-base pair stepping of DNA processing enzymes [89,90], whereas the high temporal resolution recently reported [91] enables the detection of interactions in the order of few tens of microseconds together with their sequence dependence. Such recent developments are illustrated in more detail below.

The capability to apply forces by OT has been further expanded to allow the application and measurement of torques, which has proved a great advantage in the study of DNA supercoiling. Torques can be generated and measured using magnetic tweezers, as discussed below, or through pure optical methods, for example exploiting the interaction of the trapping light with birefringent particles, as in the “optical torque wrench” [92–94].

### 2.3. Magnetic Tweezers

**Figure 4:**
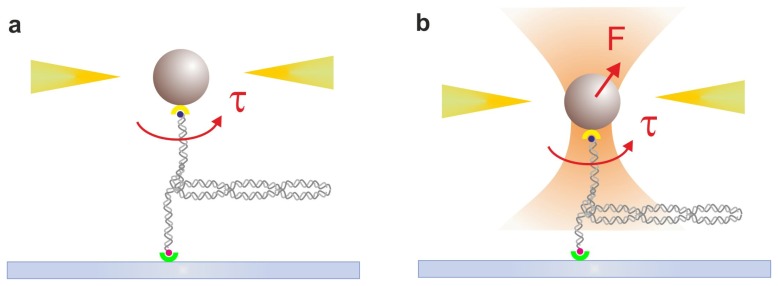
(**a**) Magnetic tweezers. A superparamagnetic bead is attached to the sample surface by a single DNA and it experiences a force due to a magnetic field along the axial direction produced by a pair of small permanent magnets (yellow). Rotation of the magnets produces torque on the magnetic bead (τ, red arrow) and consequently on the DNA molecule attached to the slide. (**b**) Magnetic tweezers combined with optical tweezers allow for independent control of force (F) and torque (τ). The figure illustrates a typical experiment performed on DNA at low applied forces; in this situation, the application of torque leads to the formation of plectonemes in the DNA molecule, as shown. Drawings not to scale.

Magnetic tweezers (MTs) provide a stable platform for measuring slow molecular processes involving both force and torque. As illustrated in Figure 4, in a MT setup a pair of permanent magnets or electromagnets is placed above the sample holder of an inverted microscope. The DNA is tethered to the surface and tagged with a magnetic bead, so it experiences an upward force proportional to the magnetic field gradient. The torque is applied by rotating the magnetic field. Due to the fine control of the tension applied and both positive and negative twists (*i.e.*, supercoils) accumulated on the DNA molecule, MTs are well-suited to study DNA topology (for a recent review see [95]) and also enzyme-catalyzed mechanisms that modulate DNA supercoiling, mainly performed *in vivo* by Topoisomerases [96,97]. Several groups implemented MTs to characterize the effect of supercoiling in diverse cellular processes. Strick and coworkers analyzed the mechanical effect of DNA supercoiling in DNA unwinding by *E. coli* RNAp, promoter clearance and recycling during transcription initiation [98], as well as the kinetics of bacterial transcription-coupled repair initiation [99]. MTs were also applied to probe the translocation onto DNA of type I restriction enzymes, to study the effect of protein processivity, loop formation and site-specific cleavage as a function of force and torsion on DNA [100]. The kinetics of LacI-DNA-mediated loop formation and structural conformations of the complex depending on DNA supercoiling were also investigated through MT assay [101].

As detailed below, one of the issues regarding the movement of proteins along DNA is to understand how the protein contends with the double-helical structure. In principle, proteins might rotate relative to the DNA while moving linearly to track the phosphate backbone. Therefore, when a protein is prevented from rotating along the helical axis of the DNA, the DNA is forced to rotate, which creates an excess of positive rotation (*i.e.*, supercoils) ahead of the protein, and an identical number of negative supercoils behind [97]. Several biological factors can prevent the rotation of proteins. For example, FtsK, a bacterial protein with DNA translocation activity used to transport chromosomal DNA during the late stages of cell division, is anchored to the cell membrane [102]. Saleh and colleagues employed MT to quantify the DNA rotation, observing ~0.07 supercoils induced into the DNA for each helical pitch traveled during translocation of the FtsK complex [103]. In addition, a bacterial transcribing RNAp can be impeded from rotating around DNA due to the nascent RNA chain, ribosomes on the mRNA, or even the growing peptide that might insert itself into the cell membrane, causing DNA to twist [97,104].

A wide array of new MT implementations has been introduced in recent years. A combined configuration of optical and low-gradient magnetic tweezers allows separate application of force and torque [105], which is highly advantageous to decouple the two effects resolution. Another important development is the possibility to directly measure torque with high on the order of approximately 1 pN·nm [106–108], analogous to the possibility to measure force in OTs. Recently, torque-clamp configurations have been introduced [109,110].

The construction of a MT setup is relatively simple compared to the complexity of an OT apparatus. Another advantage of MTs is that they act, by their very nature, as a force-clamp device. For example, van Noort and colleagues used MTs as a force-clamp to measure the stiffness and extension of single 30-nm chromatin fibers under physiological conditions [111], Moreover, MTs allow experiments on many molecules in parallel. On the other hand, OTs allow more flexibility in the experimental configuration, for example with multiple optical tweezers and the combination with fluid flow and torque. Moreover, sub-nm resolution [89,90] and microsecond temporal resolution [91], as achieved in OTs, has not been demonstrated for MTs up to now [112]. Several articles describe technical aspects of the MT setup and measurements [112–114]. As a final consideration, commercial solutions are available for MTs.

### 2.4. DNA Hydrodynamic Stretching and Curtains

**Figure 5:**
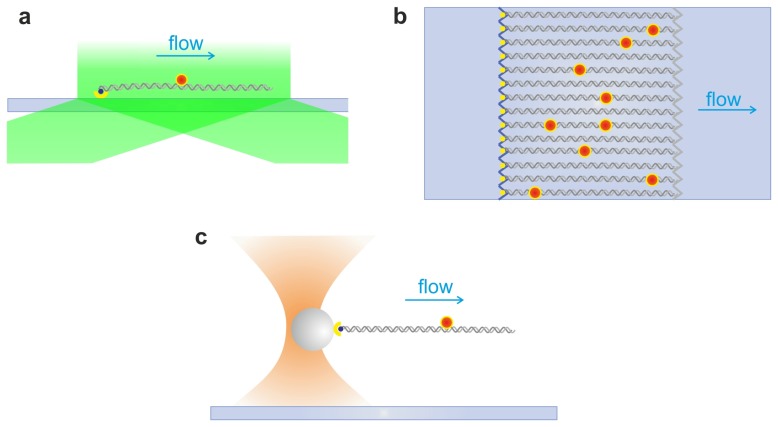
DNA flow stretching assays. (**a**) Flow-stretched DNA. A DNA molecule is attached to the surface through avidin-biotin linkage chemistry, and elongated by a shear flow (arrow). A labeled protein interacting with DNA (red dot) can be excited through an evanescent wave (green). (**b**) DNA Curtains. Several DNA molecules are aligned at a diffusion barrier (blue zig-zag line) etched in the slide surface. Upon application of the buffer flow, the DNA molecules are stretched in parallel, forming a “curtain” of DNA. (**c**) A DNA molecule is bound to an optically trapped bead and extended by flow, while the activity of a labeled protein is probed simultaneously by fluorescence microscopy. Drawings not to scale.

Ensembles of uniformly stretched DNA constitute one of the most common means to investigate the dynamics of protein-DNA interactions. Several experimental strategies are generally used to extend and align DNA molecules, including creating a receding air–water interface (termed molecular or DNA combing [115–117]) or through hydrodynamic flow. This last method involves attaching DNA molecules, usually labeled with DNA-groove binding fluorescent dyes, to the slide surface, where they are stretched along their contour length by a shear hydrodynamic flow (Figure 5a,b). It should be noted that the flow can be continuously applied to extend DNA molecules that are attached to the coverslip by a single extremity, or, alternatively, deployed to allow the specific binding in both DNA extremities. Special surface-attachment chemistries have been developed to increase the throughput of this methodology by anchoring the DNA molecules onto a fluid substrate (for example, a lipid bilayer), allowing their transverse diffusion. A diffusion barrier etched on the microscope slide causes the alignment of the molecules moving under a buffer flow [118]. This method, known as DNA curtains, allows imaging of many DNA molecules in parallel (Figure 5b).

Due to the proximity of the samples to the surface, these flow-stretching arrangements are well suited to be combined with TIRF microscopy. In this illumination strategy, only the fluorophores confined at the interface of the coverslip and aqueous buffer are excited by an evanescent wave (Figure 5a), which confers a spatial selection of the excitation volume and a resulting increase in the signal-to-noise ratio. One feature described by flow stretched DNA and TIRF microscopy was the occupancy of binding sites by LacI. Using a bacteriophage lambda DNA containing 256 tandem copies of *lac* operator, Wang and colleagues quantified the number of bound LacI-GFP molecules using an integrated photon molecular counting (IPMC) method. Their results show a mean occupancy of only 2.5% of the available operator sites, suggesting mutual exclusion effects [119]. By tracking a single LacI-GFP while diffusing along non-specific DNA [24], they also measured the 1D diffusion coefficient of the protein; the coefficient showed a broad distribution, ranging from 2.3 × 10^−4^ to 1.3 × 10^−1^ μm^2^ s^−1^. Given the large sequence variance in the non-specific region of lambda DNA, this result suggested a correlation between diffusion coefficient and the local sequence.

Besides LacI [24,120], DNA flow stretching assays were extensively used to monitor the diffusion of a wide variety of proteins along non cognate DNA such as polymerases [116,121], DNA-repair proteins [122,123], tumor suppressor protein p53 [124,125], Rad51 involved in homologous recombination [126], restriction enzymes [127], generally reporting diffusion coefficients in the order of 0.5 μm^2^ s^−1^ or smaller. These measured diffusion coefficients are consistent with a rotation-coupled sliding model [128], implying that the protein tracks the helical axis of the DNA, thus maintaining a continuous contact with DNA as it slides back and forth. Blainey and colleagues also reported rotation-coupled diffusion of the above set of DNA-binding proteins on a flow-stretched bacteriophage lambda DNA [129]. Moreover, they reported free-energy average barriers during sliding of 1.1 ± 0.2 *k*_B_*T* for proteins that have evolved in their functions for fast DNA scanning, in perfect agreement with theoretical studies [23]. Nevertheless, rotation was not directly observed by Blainey and colleagues due to the limited temporal and spatial resolution of the measurement. It was instead indirectly determined through the fit of 1D diffusion data, taking into account the protein hydrodynamic friction, and thus the dependence of *D*_1D_ on protein size [129].

Beyond 1D sliding, 3D translocations (*i.e.*, hopping and jumping) of endonuclease EcoRV along a flow-stretched DNA were directly observed by TIRF microscopy [127]. Fast and large translocation steps of greater than 200 nm between two consecutive frames (at 20 ms integration time) were observed and attributed to jumping events. An increase of salt concentration to physiological conditions led to a strong reduction of the jumping frequency and increased interaction time for EcoRV. Indeed, the buffer conditions used in single-molecule experiments, namely non-physiological salt concentrations, often raise concerns about the ability of these experiments to accurately assess the behavior of DNA-binding proteins in the cell. Addressing this issue is of crucial importance. It should be highlighted that many single-molecule imaging measurements are limited in their time resolution due to the exposure times necessary to obtain an adequate signal for single-molecule detection and tracking. In these conditions, rapid events of dissociation, 3D diffusion and re-association occurring between the recorded frames cannot be excluded *a priori*. These limitations impose caution on interpreting the data directly as 1D diffusion of the protein along the DNA. However, since pure 1D protein sliding should not depend on salt concentration, repeating the measurements at different ionic strengths provides an estimate of the contribution from 3D diffusion: an increased coefficient at higher salt concentrations is consistent with hopping/jumping events. In the experiments by Bonnet *et al.*, the distance covered by sliding was reduced at higher salt concentration, while the distance covered by jumping was roughly unchanged [127]. These experimental results are in good agreement with theoretical models that characterize the 1D and 3D contributions to a facilitated diffusion mechanism [130,131]. Recently, a similar experimental setup was used to characterize the rotation-coupled sliding of EcoRV as a function of varying sizes of the conjugated fluorescent labels [132]. Organic dyes, fluorescent proteins and Quantum dots were used to label EcoRV, and a significant dependence of the measured *D*_1D_ coefficients on the size of the label was observed.

Another recent study explores the contribution of hopping and jumping to the capacity for bypassing roadblocks [133]. This study represents one of the few *in vitro* single-molecule experiments to address the question of molecular crowding and the presence of roadblocks along DNA, which are expected to hinder 1D sliding and increase viscosity and target searching time. The potential to obstruct protein mobility gains even more relevance in eukaryotic cells, where most DNA-binding proteins such as transcription factors and DNA repair proteins cannot mechanically disrupt nucleosomes to move along chromatin, in contrast to DNA translocases [134]. Gorman and colleagues found that some components of the post-replicative mismatch repair proteins, namely Mlh1-Pms1, are able to bypass nucleosomes and protein roadblocks through hopping and jumping. These experimental results led to the conclusion that 1D sliding can occur on crowded DNA molecules, although the ability to bypass obstacles depends on the 1D diffusion mechanism along DNA [133]. According to their observations, only proteins experiencing rotation-coupled sliding overcome barriers by 3D steps, while proteins that do not track the phosphate backbone remained trapped between nucleosomes. With an extended DNA configuration it is thus possible to characterize the dynamics of protein-DNA interactions at the single-molecule level. By localizing and tracking a single fluorescently tagged protein, specifically bound to DNA or undergoing 1D diffusion, it is possible to measure binding and unbinding kinetics of single proteins to specific and/or non-specific DNA sequences together with 1D diffusion coefficients.

### 2.5. Single Optical Tweezers and Flow Extended DNA

The behavior of individual proteins that bind to and/or translocate on DNA has been widely studied by the Kowalczykowski’s group, mainly in the field of recombinational DNA repair [135]. The experimental strategy combines optical trapping to capture a DNA molecule, which is subsequently extended with a flow, with single-molecule fluorescence microscopy to image labeled repair proteins and/or DNA (Figure 5c). Initially, Bianco *et al.* [136] measured the translocation velocity of RecBCD by monitoring the enzyme-induced displacement of a fluorescent DNA intercalating dye. RecBCD processively unwound 42,300 bp of double-stranded DNA at a maximum rate of 972 ± 172 bp/s. Subsequently, the authors studied the interaction of RecBCD with an octameric DNA sequence called Chi (Crossover hotspot investigator), involved in the regulation of homologous recombination [137]. Spies and colleagues showed that the interaction with the Chi-sequence affected RecBCD translocation. Upon Chi recognition, the enzyme pauses for approximately 5 s and then translocates again at a reduced velocity, due to a switch in the lead-motor domain [138].

Notwithstanding the vast contribution to the elucidation of protein-DNA interactions and to the current understanding of how proteins locate their targets, the flow-extended DNA experiments highlighted in sections 2.4 and 2.5 present some drawbacks mainly regarding their limited control on DNA extension. In these assays, the DNA end-to-end distance is usually determined by imaging intercalating fluorescent dyes, which are sensitive to photobleaching and have the potential to affect protein mobility. Moreover, the continuous buffer flow required to elongate DNA, can bias protein motion [126], especially when DNA is anchored at only one end. Furthermore, regarding the surface-tethered assays presented in section 2.4, care must be taken in selecting a proper surface immobilization strategy to avoid excessive sticking of the DNA to the surface. These technical challenges have been addressed in detail in several publications [117–140].

### 2.6. Dual Optical Tweezers Assays

**Figure 6:**
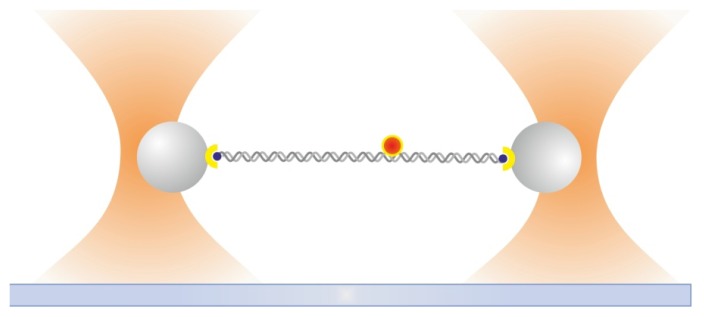
Combined dual optical tweezers (2OTs) and fluorescence assay. A single protein labeled with a fluorophore (red dot) interacting with DNA suspended between the double optical tweezers can be localized and tracked with high accuracy. Drawing not to scale.

Dual optical tweezers (2OTs) [80,81,141,142] provide a means to precisely control the end-to-end distance of single DNA molecules without requiring DNA staining. Moreover, 2OTs suspend the trapped DNA molecule in solution circumventing the problem of potential surface effects as well as the need for a continuous flow to extend the DNA. Additionally, they provide sub-nanometer stable support for the DNA molecule, free from drifts and mechanical vibrations of the microscope stage, offering improved performance in localization and tracking measurements. In a 2OT assay, a DNA molecule is tethered between two optically trapped beads in a “dumbbell” configuration (Figure 6), and its elastic properties including persistence length, bending stiffness, and contour length are measured with high precision. A variation of this geometry entails substituting one of the two OTs with a micropipette [143]. Recent advancements in optical trapping allow the measurement of sub-nanometer displacements induced by protein-DNA interactions, while simultaneously allowing for the application of controlled pN forces. The implementation of this OT assay to the study of protein-DNA interactions permitted the step-by-step monitoring of transcription by RNAp at base-pair resolution [89], the coupling between DNA unwinding and replication [144], and DNA packaging into virus capsides [145–148], among many others (see [86] for a recent review). Other landmark studies applied to nucleosomes include the recent observation of the unfolding of single nucleosomes on chromatin fibers [149], the probing of higher-order structure of single chromatin fibers according to force and ionic strength [150], and the transcription of nucleosomal DNA by RNApII [151], among others. The connection between the mechanics of DNA-target recognition, subsequent conformational changes such as bending induced upon binding, and cleavage rates by restriction enzymes as a function of DNA tension was addressed with 2OTs [152]. Later on, Wuite and coworkers also evaluated the enhancement of target recognition by restriction enzymes due to DNA coiling, in one of the few single-molecule studies addressing the relative impact of 3D searching pathways in the facilitated diffusion model [153]. The authors obtained different degrees of coiling by tuning the DNA extension with 2OTs. By acquiring specific association rates of EcoRV as a function of varied DNA conformations, Wuite and colleagues observed that the targeting rates almost doubled when the DNA configuration was manipulated from fully extended to coiled, depending on salt concentration [153].

### 2.7. Combining Dual Optical Tweezers and Single-Molecule Fluorescence Microscopy

Implementing single-molecule fluorescence detection and 2OTs in a hybrid experimental setup combines the advantages of both techniques to get an improved readout of the biochemical process under study [154,155]. Pioneering hybrid single-molecule assays combined 2OTs and TIRFM to study the movement of a Cy3-labeled RNAp diffusing along DNA template [156]. To combine OTs with the TIRFM requirement of close proximity to the surface, Yanagida and colleagues engineered coverslips with a series of pedestals and indentations to trap beads and align the DNA at the surface to allow efficient TIRF excitation. With this hybrid assay they achieved the first direct observation of RNAp sliding along DNA, and a concomitant measurement of the enzyme affinity for specific promoter sites and non-specific sequences [156]. Furthermore, they visualized an enhancement of RNAp binding to relaxed DNA (*i.e.*, not stretched to its total contour length). The authors obtained an estimated diffusion coefficient one to three orders of magnitude smaller than those predicted by biochemical studies, possibly due to their limited spatial resolution of approximately 200 nm, or about 600 bp.

Wide-field epi-illuminated fluorescence microscopy permit complete surface decoupling when conjugated with 2OTs in a dumbbell configuration (Figure 6) [155]. The potential of this technique can be illustrated by the investigation of the interaction dynamics of DNA-Rad51 [157], a nucleoprotein involved in DNA repair through homologous recombination. By quantifying the fluorescence arising from Rad51 patches with high accuracy, van Mameren and colleagues could count the number of Rad51 monomers interacting with DNA and dissect the mechanistic model of Rad51 disassembly. The disassembly process resulting from the interplay between tension-independent ATP hydrolysis and the release of the tension stored in the filament at a force-dependent rate could only be revealed thanks to the simultaneous monitoring of the fluorescence signal from the protein and the tension of the DNA [157].

Another recent study reports the combination of single-molecule fluorescence and 2OTs to directly visualize the sliding of restriction enzyme EcoRV labeled with a single Quantum dot (QD) along a DNA molecule held by 2OTs [158]. QD nanocrystals are very bright and photostable fluorescent probes, allowing higher localization accuracy in comparison to single organic fluorophores [155,159]. Indeed, Biebricher and coworkers could track individual enzymes at varying levels of DNA stretching for tens of seconds with 40 ms integration time and 30 nm spatial resolution without the limitation of photobleaching effects. The diffusion coefficients, however, were determined to be on the order of ~3 × 10^−3^ μm^2^ s^−1^, considerably reduced compared to dye-labeled EcoRV. This effect may be due to the larger hydrodynamic radius of QDs with respect to the enzyme and conventional organic fluorophores [132,158]. QDs also exhibit an intermittency phenomenon (blinking) as they undergo transitions between fluorescent and non-fluorescent states, which can deteriorate their quantum yield. On the other hand, blinking can be a useful property to distinguish a single QD from multiple QDs or aggregates. Care must be taken with the use of QDs because of the potential to alter protein activity or native diffusion behaviour. In Biebricher *et al.*, QD labeling did not affect the biochemical activity of the enzyme, as confirmed by direct observation of DNA cleavage on an elongated DNA strand tethered to the surface. Moreover, they had strong indications that a slight overstretching of the DNA, at a 5% increase over its contour length, led to a significant decrease of the measured diffusion coefficient *D*_1D_, suggesting a subsequent change in the energetic landscape of sliding [158].

One of the biggest challenges in combining OTs and fluorescence microscopy is the dramatic reduction in fluorescence longevity due to the coincident irradiation with OT and excitation beams [160]. The resulting enhanced photobleaching is due to the exposure of excited-state fluorophores to the high photon flux of the OT beam. The aforementioned studies circumvent such limitations by adopting a spatial separation of the fluorophore from the trapping beam, which extends the fluorescence signal of organic fluorophores for an average of 30 s [154]. Indeed, several micrometer-long DNA molecules are commonly used, for example the lambda bacteriophage DNA that consists of approximately 48 kbp of double-stranded linear DNA with 12-nucleotide single stranded segments at both 5′ ends facilitating end-labeling with modified deoxynucleotides [161]. An alternative to these spatially based separation approaches is temporal separation, obtained by alternating fluorescence excitation and optical trapping lasers [162]. These approaches, which have been applied to monitor DNA unzipping [162] and oligonucleotide hybridization [163] are equally useful to improve fluorescence longevity when coincident trapping and fluorescence excitation are required.

Localization Accuracy of DNA-Bound Fluorescent Proteins

The ability to resolve the position of an individual fluorescence emitter with high accuracy is well established. Imaging a fluorophore reveals a point source with a finite Airy disk point spread function (PSF). The center of mass of such a distribution represents the position of the fluorophore and can be determined with much higher precision than its width by performing a fit of an appropriate function, usually a two-dimensional Gaussian [164–167]. The localization accuracy is limited by the number of photons detected (*N*), the size of the detector pixel (*a*), the width of the PSF (*s*), and the background noise (*b*). Thompson *et al.* derived a relationship to determine the uncertainty (σ_μ_) in the localization of the fluorophore [168]:

(1) $$\sigma_{\mu }=\sqrt{\left(\frac{s^{2}}{N}+\frac{a^{2}/12}{N}+\frac{8\pi s^{2}b^{2}}{a^{2}N^{2}}\right)}$$

The first term in the equation represents the optical resolution of the microscope, the second term reflects the increase in the error due to the finite pixel size of the detector, whereas the last term takes into account the effect of background noise. One of the first successful implementations of single-molecule imaging with high localization accuracy was obtained in Selvin’s lab to directly visualize the stepping mechanism of myosin V [169]. Through fluorescence imaging with one nanometer accuracy (FIONA), Yildiz *et al.* tracked the movement of myosin V labeled with an organic dye along surface-immobilized actin filaments with ~1.5 nm position accuracy and 500 ms integration time [169].

In the dual trapping and fluorescence hybrid single-molecule assays described above, the localization precision of single, fluorescently labeled, DNA-bound proteins is further affected by thermal fluctuations. DNA is a semiflexible polymer with a persistence length of ~150 bp at physiological conditions. The long molecules employed in the dumbbell experiments, therefore, exhibit thermal fluctuations that depend on the tension applied to the molecule and are not usually negligible on the scale of nanometric protein localization. Candelli *et al.* have recently addressed the capability of resolving the position of fluorescently labeled proteins as a function of DNA mechanical fluctuations at different end-to-end distances [154]. As the DNA molecule is stretched, the width of the imaged spot (*s_x_*) decreased from *s_x_* = 405 nm at tension << 1 pN to *s_x_* ~ 150 nm at tensions above few pN, thus yielding localization accuracies strongly dependent on applied tension (σ_μ_*_x_~* 200 nm for *F* < 0.1 pN and σ_μ_*_x_* ≤ 10 nm to tensions above 1 pN). These measurements, performed with 1 s integration time, are in agreement with theoretical predictions of DNA thermal fluctuations using the equipartition theorem, showing localization accuracies limited only by the diffraction limit at tensions greater than 1 pN [154].

The localization of single molecules by fluorescence imaging demonstrates an intrinsic compromise between localization accuracy and temporal resolution. As discussed above, high localization accuracy requires a high number of photons collected (Equation 1), and thus a slow acquisition rate. Therefore, to overcome this limitation, a different experimental configuration must be conceived.

### 2.8. Ultrafast Force-Clamp Spectroscopy

**Figure 7:**
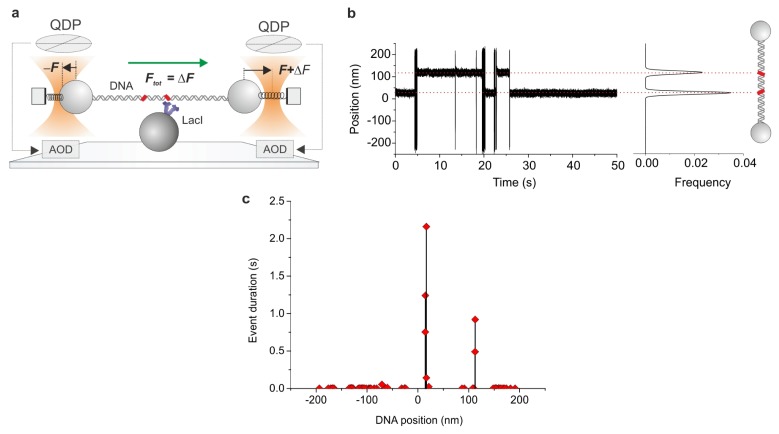
Ultrafast force-clamp spectroscopy. (**a**) A constant force *F_tot_* = Δ*F* is applied to DNA suspended between the 2OTs through two feedback systems clamping the force on the left and right bead to −*F* and *F* + ΔF, respectively. The force is measured using Quadrant Detector Photodiodes (QDP) and kept constant by moving the traps through Acousto-Optic Deflectors (AOD). The dumbbell is then brought into the proximity of a third bead attached to the microscope coverslip to allow LacI (blue) interaction with DNA. Red areas represent the two operator sequences which are separated by ~300 bp. (**b**) On the left, 50 s of a typical record of a LacI molecule interacting with DNA (*F_tot_* = 5 pN, ±200 nm confined dumbbell oscillation). Distribution of bound positions shows two peaks separated by ~96 nm, in agreement with our interoperator distance. (**c**) The duration of interaction events versus relative position on the DNA shows long events corresponding to the two operators, while all other durations are representative of short interactions with non-specific DNA. Drawings not to scale. Adapted by permission from Macmillan Publishers Ltd: (Nature Methods) [91], copyright (2012).

We recently developed a purely mechanical approach called ultrafast force-clamp spectroscopy to probe interactions between proteins and DNA [91]. Our system is based on a dual-trap force-clamp configuration and is capable to apply constant loads between a binding protein and an intermittently interacting DNA molecule (or another polymer, such as actin or a microtubule). A sketch of the operational principle of our method is shown in Figure 7a. Briefly, an alternated force is applied to the dumbbell, resulting in a triangular wave movement when DNA is not bound to the protein. Upon interaction of DNA with the surface immobilized protein, the load is rapidly (~10 μs) transferred to the protein and the dumbbell movement stops, thus revealing the protein interaction. The method allows detection of interactions as brief as ~100 μs and probes sub-nanometer conformational changes with a time resolution of tens of microseconds. With this method we were able to measure the sequence- and force-dependence of LacI binding to DNA, dissecting two kinetically distinct dissociation rates, and characterize LacI interactions with specific operator and non-specific DNA sequences (Figure 7b,c) [91]. The unprecedented time resolution of the method and the capability to reveal conformational changes of the interacting proteins promise to shed new light on the mechanisms of target search and recognition on DNA, as well as the force-dependence of protein-DNA interactions and gene regulation, and the fast kinetics potentially involved in genetic noise and stochastic transcription events.

## 3. Probing Facilitated Diffusion and Protein-DNA Interaction Dynamics *In Vivo*

Most *in vitro* single-molecule strategies reported here and in the literature characterize protein-DNA interactions and target search mechanisms under conditions that are often quite far from physiological. This is understandably due to the required decrease in the level of complexity for *in vitro* assays and to the limitations of current single-molecule techniques. For example, elongated DNA molecules allow for easier localization and visualization of fluorescently labeled proteins but restrict DNA coiled conformations naturally occurring *in vivo*. Often low-salt buffers are used to promote DNA binding at the nM protein concentration regime required for single-molecule detection, which can also lead to longer trajectories and residence times. Additionally, diffusion coefficients are expected to depend on salt concentration when a hopping component is involved in the translocation mechanism, because high salt concentrations promote dissociation events from the DNA [122,127]. Likewise, protein concentration may play a key role in facilitating the removal and renewal of proteins bound to the DNA, as shown for *E. coli* proteins involved in architectural organization and transcriptional regulation [170]. Also the effect of both 1D and 3D macromolecular crowding which, respectively, hinders protein sliding and increases the viscosity by at least tenfold [171], is not often explored by single-molecule *in vitro* assays. Therefore, despite the outstanding technical improvements obtained in the last decades and the enormous contributions of the assays described above, it is still being debated as to what extent the measured parameters including sliding lengths, interaction kinetics, and the effects of DNA occupancy reflect the equivalent properties *in vivo*.

The main challenge of probing protein-DNA interactions *in vivo* relies on overcoming the strong cellular autofluorescence background and the dispersion of the fluorescence signal throughout the whole cell arising from freely diffusing fluorescent molecules. Thus, common illumination strategies aim to reduce the detection volume to decrease noise from out-of-focus fluorescence and/or to perform 3D sectioning with a reasonable temporal resolution to image the whole cell body [5].

The patterns of protein mobility *in vivo* at the level of the single cell have been widely assessed through Fluorescence Recovery after Photobleaching (FRAP) and Fluorescence Correlation Spectroscopy (FCS). In FRAP [172], a finite region is depleted of fluorescence by exposure to an intensely focused laser beam, and the subsequent recovery of fluorescence due to trafficking or diffusion of tagged protein into the photobleached area is recorded. FRAP is well suited to probe the diffusive properties [173] and binding [174] of fluorescently tagged proteins; the method inherently lacks single-molecule sensitivity. In one elegant study, Phair *et al*. used FRAP to quantify the interactions of an array of nearly 20 nuclear proteins with native chromatin in intact cells [175]. The authors observed that at any given time, most of the proteins were bound to DNA, albeit showing similar short mean residence times of approximately 2 to 20 s. This finding is consistent with transient interactions with non-specific DNA, a common feature to all the chromatin-associated proteins probed by Phair *et al.*, including structural proteins, remodeling factors, transcriptional coactivators, and transcription factors. The observed dynamic exchange of chromatin-associated proteins combined with the large population of bound molecules suggested a continuous scan of the genome for appropriate binding sites by 1D sliding and 3D diffusional hopping between chromatin fibers [175]. Nonetheless, the precise quantification of any potential non-specific interactions is extremely difficult through solely FRAP measurements, because different models can lead to discrepancies in the measured parameters [176,177]. FRAP has also been used to probe promoter binding, transcription initiation and elongation rates of RNApI [178] and RNApII [179,180], among others [181].

FCS has also been applied to quantify the mobility and local concentration of proteins in living cells, by measuring the fluctuations in fluorescence intensity in a diffraction-limited spot [182–184]: the more mobile the protein, the more frequent and short-lived the fluctuations in the spot. FCS was used *in vivo* to measure the binding kinetics and residence time [185] as well as the association and dissociation rates [186] of GFP-tagged transcription factors with chromatin. Since bound and unbound states lead to FCS data that fits a model with two diffusing components, FCS can estimate the 1D and 3D diffusion components of the facilitated diffusion mechanism. The time in the unbound state corresponds to 3D diffusion within the nucleus, and the time in the bound state corresponds to 1D diffusion along DNA while the protein is non-specifically bound [120]. These properties were recently exploited by Larson and colleagues with two-photon FCS to monitor the 1D/3D diffusion of the transcription factor Mbp1 in the nucleus of yeast cells [187]. They obtained dwell times of τ_3D_ = 1.1 ± 0.2 s and τ_1D_ = 0.8 ± 0.1 s and an effective diffusion coefficient of 0.6 μm^2^ s^−1^. The estimated mean target search time was 52 s, while the single-target search time of a single Mbp1 in the yeast nucleus was about 5 h, in good agreement with their endogenous conditions of approximately 350 copies of labeled Mbp1 proteins in the nucleus [187].

**Figure 8:**
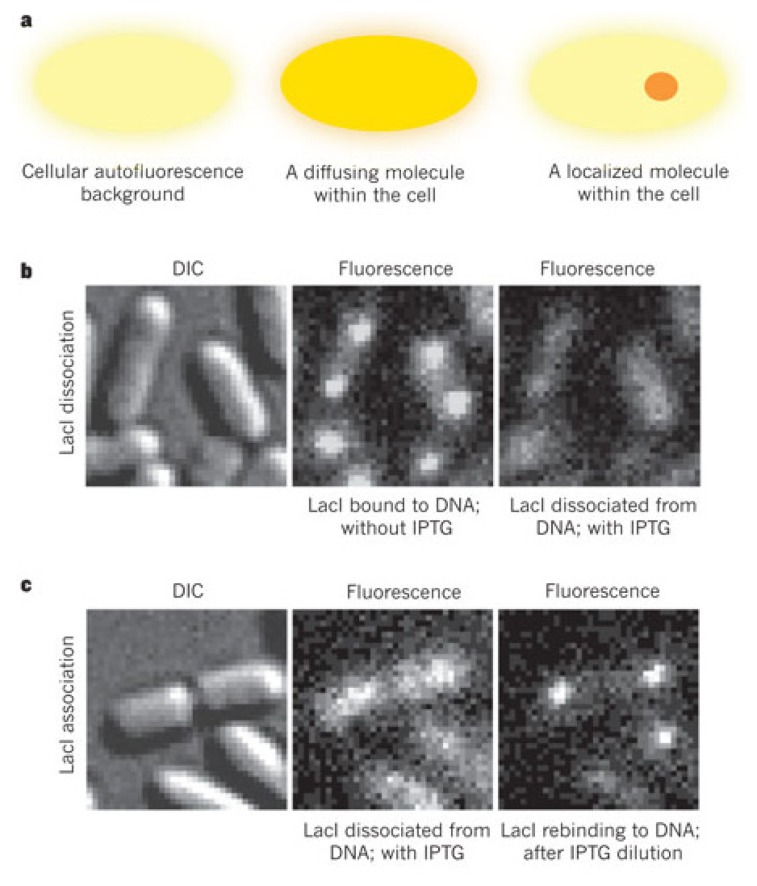
Real-time monitoring with single-molecule sensitivity of LacI dynamics in living bacteria. (**a**) Detection by localization. Freely diffusing protein is barely detectable due to the spreading of its fluorescence signal throughout the whole cell. However, when the protein is bound, its signal can be detected over the cellular autofluorescence background. LacI dissociation (**b**) and reassociation (**c**) dynamics upon addition and dilution of the inducer IPTG, respectively. DIC (differential interference contrast microscopy) and fluorescence (1 s integration time) images of bacterial cells expressing LacI-Venus. In absence of IPTG, LacI can be imaged when bound to its operator(s). The localized fluorescence disappears as a consequence of LacI dissociation from the operator upon addition of IPTG. Adapted by permission from Macmillan Publishers Ltd: (Nature) [188], copyright (2011).

Sunney Xie’s group made a breakthrough step towards the *in vivo* probing of facilitated diffusion mechanisms and gene expression at single-molecule level, demonstrating with the lac operon an example of stochastic gene expression and correlating stochastic fluctuations with the binding behavior of LacI to DNA [120,188,189]. Their living cell assay allowed the measurement of transcription factor-DNA interactions in an *E. coli* strain expressing LacI dimers fused at the C-terminal with a fast-maturing (~7 min) yellow fluorescent protein (YFP) Venus [120]. When bound to its operator in the relatively stationary *lac* operon, LacI-Venus could be imaged as a diffraction-limited spot because the localized fluorescence could be detected above the autofluorescence background (Figure 8). Upon addition of the inducer isopropyl-β-d-thiogalactoside (IPTG), LacI dissociates and thus the localized fluorescent foci disappear. These observations allowed Elf *et al.* to measure the kinetics of binding and dissociation of LacI to the operator in response to environmental signals. The search time of a single LacI to reach a vacant operator was about 270 s, with an estimated mean duration of non-specific interactions of about 5 ms [120]. Moreover, through stroboscopic laser excitation and Single-Particle Tracking (SPT) analysis of individual trajectories, the effective diffusion coefficient of LacI *in vivo* was measured as 0.4 μm^2^ s^−1^, considerably higher than the *D*_1D_ ~0.05 μm^2^ s^−1^ obtained in their *in vitro* assay performed on flow-extended DNA. The authors interpreted the higher diffusion coefficient obtained *in vivo* as a consequence of the facilitated diffusion of the protein to locate the operator sequences. The authors implemented FCS to determine the 3D diffusion of a LacI mutant lacking the DNA-binding domain. The evaluated 3D diffusion coefficient was *D*_3D_ ~3 μm^2^ s^−1^, which led to the estimation that LacI spends 87% of the time sliding along non-specific DNA and only 13% freely diffusing in the cytoplasm [120].

Elf and colleagues obtained the first direct experimental observation of LacI facilitated diffusion to locate the operator sequences in living bacterial cells with single-molecule sensitivity [22], with a similar imaging assay as described above [120]. The authors measured the search time of LacI-Venus dimers based on the distinction between localized and diffusive fluorescence signals with improved accuracy by carefully ensuring a low number of labeled proteins. To that end, Hammar *et al.* engineered a strong autorepressor system to limit the number of LacI per cell and to maintain a low and even expression of LacI-Venus at 3 to 5 dimers per cell. The association rate of LacI to the individual operator site was thus determined after removing the inducer IPTG. The authors found an average search time of about 56 s, corresponding to an approximate time of 3–5 min required for a single repressor dimer to bind the operator. To directly determine the sliding length of LacI along non-specific DNA sequences *in vivo*, Hammar *et al.* made several *E. coli* strains containing two identical operator sequences at different interoperator distances, similar to the bulk *in vitro* assays [190]. Briefly, if the sliding distance is smaller than the interoperator distance, the two operators are perceived as two independent targets in the search process, and vice-versa. Hammar and colleagues observed two distinct binding events when the operators were separated by 105 bp or more, and estimate an effective sliding distance of approximately 45 ± 10 bp [22]. To address the effect of roadblocks on protein sliding along DNA, Hammar and colleagues placed a different repressor operator (TetR) close to the single lac operator. They observed a reduced binding rate of LacI by a factor of 1.75 in the presence of TetR protein, suggesting that sliding along DNA can be obstructed by other DNA-bound proteins. Finally, from these results, the authors predicted a low probability of binding to the operator, demonstrating that LacI spends greater than 90% of the time interacting non-specifically with DNA and sliding over the operator several times before binding [22].

## 4. Conclusions

The mechanisms of protein-DNA interactions have been investigated for many years using a variety of microscopy techniques, but questions remain. The importance of these processes in the normal and pathological workings of the cell warrants the widespread interest in all methods suitable for probing protein-DNA interactions and their dynamics. In this review, we have provided a description of the single-molecule methods recently developed in this research field. The extension of these methods from the *in vitro* to the *in vivo* realm has already demonstrated great potential and will undoubtedly represent one of the most exciting areas of technological development for the study of protein-DNA interactions.
